# The Impact of ZnO Nanofillers on the Mechanical and Anti-Corrosion Performances of Epoxy Composites

**DOI:** 10.3390/polym16142054

**Published:** 2024-07-18

**Authors:** Raluca Şomoghi, Augustin Semenescu, Vili Pasăre, Oana Roxana Chivu, Dan Florin Nițoi, Dragoş Florin Marcu, Bogdan Florea

**Affiliations:** 1 Faculty of Petroleum Refining and Petrochemistry, Petroleum-Gas University of Ploiesti, 100680 Ploiesti, Romania; 2 National Institute for Research and Development in Chemistry and Petrochemistry—ICECHIM, Splaiul Independentei Street, No. 202, 6th District, 060021 Bucharest, Romania; 3 Faculty of Materials Science and Engineering, National University of Science and Technology POLITEHNICA Bucharest, Splaiul Independentei Street, No. 313, 6th District, 060042 Bucharest, Romania; dragos.marcu@upb.ro (D.F.M.); bogdan.florea1411@upb.ro (B.F.); 4 Academy of Romanian Scientists, 3 Ilfov Str., 5th District, 050044 Bucharest, Romania; 5 Faculty of Industrial Engineering and Robotics, National University of Science and Technology POLITEHNICA Bucharest, Splaiul Independentei Street, No. 313, 6th District, 060042 Bucharest, Romania; oana.chivu@upb.ro (O.R.C.); dan.nitoi@upb.ro (D.F.N.)

**Keywords:** ZnO, epoxy resin, mechanical property, anti-corrosion

## Abstract

Epoxy resins were reinforced with different ZnO nanofillers (commercial ZnO nanoparticles (ZnO NPs), recycled ZnO and functionalized ZnO NPs) in order to obtain ZnO–epoxy composites with suitable mechanical properties, high adhesion strength, and good resistance to corrosion. The final properties of ZnO–epoxy composites depend on several factors, such as the type and contents of nanofillers, the epoxy resin type, curing agent, and preparation methods. This paper aims to review the preparation methods, mechanical and anti-corrosion performance, and applications of ZnO–epoxy composites. The epoxy–ZnO composites are demonstrated to be valuable materials for a wide range of applications, including the development of anti-corrosion and UV-protective coatings, for adhesives and the chemical industry, or for use in building materials or electronics.

## 1. Introduction

**Figure 1:**
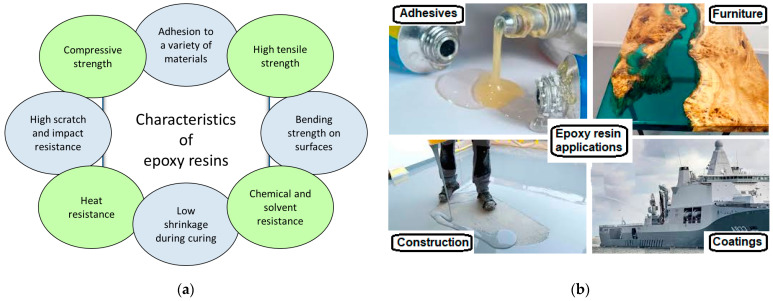
The schematic representation of (**a**) the main characteristics of epoxy resins; (**b**) the applications of epoxy resins.

Epoxy resins are thermoset resins that typically comprise four ingredients: the monomer resin, a hardener or crosslinking agent, an accelerator, and a plasticizer. Epoxy resins are versatile and indispensable in modern materials due to their adhesive properties regarding many materials (i.e., wood, stone, fibers, metals, ceramics, or plastics) [1,2,3,4], good mechanical properties (such as tensile strength, flexibility, compression resistance, and ductility) [5,6,7], great chemical resistance (particularly in alkaline environments) [8,9], high thermal stability [10], low shrinkage during curing, and low curing time (Figure 1a).

Various types of epoxy resins are available (i.e., bisphenol A (DGEBA), cycloaliphatic epoxy resin, resole resin, and waterborne epoxy resin), and can be utilized in a wide range of applications such as adhesives, encapsulated materials, mechanical reinforcement, coatings, thermoset composites, constructions, aerospace and electronics industries, and automotive systems [11,12,13,14] (Figure 1b). These epoxy resins present different epoxy rings, namely, (1) aromatic epoxy saturated rings (called aliphatic) and (2) nonaromatic saturated ring epoxy (called cycloaliphatic). When the aromatic rings are present on the aliphatic epoxy, the resistance of the epoxy to ultraviolet radiation is enhanced. In this case, this type of epoxy resin can be used for outdoor applications [15]. The conventional difunctional epoxy is utilized in matrices of composite materials [16]. High-performance applications (tensile strength, high viscosity, heat resistance and higher thermal stability) require the use of multifunctional epoxies (epoxy with higher functionality) [17,18]. On the other hand, the utilization of epoxy resin has some disadvantages, as well, including low elasticity [19], low UV ray resistance [20], and lower long-term corrosion prevention [21]. When the epoxy resin is mixed with the hardener in order to achieve the epoxy coating, the emission of volatile organic compounds occurs during the curing process, contributing to air pollution. Exposure to such compounds can lead to several health issues (i.e., skin irritation and headaches).

The epoxy resin’s role in the material composite is to send the force equally to the filler and protect the whole composite’s system integrity [22]. Epoxy composites are inherently brittle due to their high crosslinking capacity, which lead to low resistance to crack initiation and growth [23]. The most common key goal among researchers is to make epoxy composites with a high level of hardness without compromising the essential characteristics (thermo-physical and mechanical characteristics), which are highly important properties in several applications, such as construction and coatings [24,25,26].

Epoxy coating is commonly used because of its good corrosion resistance, chemical resistance, and adhesion, and its anti-corrosion performance can be superior to that of metals [27,28]. Epoxy coatings are hydrophilic in nature, possess high-volume shrinkage upon curing, and can absorb water from the environment. Due to the defects of the epoxy coating, corrosive-medium molecules (i.e., water, oxygen, and chloride anions) can penetrate the epoxy coating, thereby reducing the anti-corrosion performance. Zhou et al. [29] studied the properties of epoxy composite coatings, like adhesion and corrosion resistance. The results indicated that the acrylate copolymers, synthesized via radical polymerization, have strong hydrogen bond networks and present good dispersion in epoxy composites, which improves the adhesion and anticorrosion performance of epoxy coating. Appusamy et al. [30] prepared epoxy composites through the 3D printing technique, presenting excellent mechanical properties (good flexibility and hardness). Marichelvam et al. [31] examined the mechanical performances of obtained hybrid composites using various fibers and epoxy resin. The final results showed that the samples exhibited good tensile strength, bending strength, hardness and impact resistance, and can be used as a potential material to reinforce the concrete composites.

Many studies have shown that the incorporation of nanofillers (i.e., zinc oxide (ZnO), titanium dioxide (TiO_2_), alumina (Al_2_O_3_), silicon dioxide (SiO_2_), or carbon nanotubes (CNTs)) can significantly adjust the viscosity of the resin, acting as hardening agents and limiting the flexibility and mobility of the epoxy chains [32,33,34,35]. These nanofillers can be dispersed in the epoxy matrix to produce epoxy coatings with good thermal resistance [36,37], superior mechanical properties, and increased resistance in corrosive environments [38,39,40].

ZnO nanoparticles (ZnO NPs) are commonly used as nanofillers in the epoxy matrix to improve the electrical and thermo-mechanical properties of nanocomposites due to their thermal conductivity, high melting temperature, and hardness [41].

ZnO nanofillers have attracted enormous attention from the scientific community as an anti-corrosive material compared to other nanofillers due to their characteristics, such as non-toxicity, UV degradation resistance, corrosion inhibition properties, and environmentally friendly nature [42,43]. However, the amount of ZnO nanofillers used must be carefully selected, in order to avoid the formation of large ZnO agglomerates. Several studies have suggested that uniform and less rough epoxy coatings can be obtained if a homogeneous dispersion of ZnO NPs is used [44,45]. It has been observed that the size and morphology of ZnO (both commercially available ZnO and functionalized ZnO) significantly influences the physical and chemical characteristics of the final nanocomposites [46,47,48]. Different types of structures based on ZnO, with various morphologies, are presented in the literature [49,50] and obtained using commercial ZnO NPs and ZnO NPs functionalized with other materials (e.g., graphene oxide (GO), graphene (Gr), silane agents ((3-glycidyloxypropyl)trimethoxysilane (GPTMS), octadecyl triethoxysilane (ODTES)), titanium dioxide (TiO_2_), and cupric oxide (CuO)) [43,49,51,52,53,54,55,56]. Among the advantages of this particular approach can be listed the enhanced shielding capacity against UV radiation, enhanced dispersion, and the reduced agglomeration of nanoparticles, leading to the improvement of the surface-active sites and an increase in the chemical stability, as well as superior compatibility with the epoxy resin matrix [48,49,50,51,52,53,54,55,56]. These ZnO nanocomposites were prepared using different processing methods, such as the sol–gel process, hydrothermal method, microwave-assisted method, and co-precipitation method, and were used to obtain coatings with superior self-cleaning, mechanical, and corrosion-resistance properties [49,57,58,59,60,61].

Several studies reported that the addition of ZnO NPs increases epoxy stiffness [62] and tensile strength [63] and improves resin surface damage under multiple scratch conditions after viscoelastic recovery [64,65]. A major conclusion of the studies is that the fraction of nanoparticles that can be loaded into an epoxy resin must be limited, in order not to affect the viscosity of the epoxy system [66]. Also, as a general conclusion, it was observed that the percentage of ZnO added to the epoxy resin influenced the hardness of the nanocomposite [67].

The addition of ZnO nanofillers into the epoxy matrix aims to overcome the shortcomings of epoxy coatings and improve corrosion protection. In this way, the lifetime of metal structures will increase, and maintenance and repair costs will be reduced [68]. The published studies have shown that nanoscale fillers improved the adhesion strength and the cohesive strength in epoxy-based coatings, demonstrating that the adhesion mechanisms are influenced by the types of curing agents and adhesion promoters used in the curing process [69,70,71,72].

Madhup et al. [70] investigated the cohesive and adhesive properties of the ZnO–epoxy coatings on the carbon steel surface. The composites were made using various concentrations of ZnO NPs (0, 0.25, 0.5, 1, and 2 wt. %) as nanofillers and bisphenol A as the epoxy matrix. They concluded that a concentration of 0.5 wt. % ZnO NPs provides good cohesion and adhesion over the metal surface.

Şomoghi et al. [71] evaluated the resistance to scratching of a carbon steel substrate covered with an epoxy resin composite containing ZnO NPs and halloysite nanotubes (HNT). The prepared coated materials were immersed in seawater (salinity of 1%) and subjected to the scratching tests, with the results of the study demonstrating an increase in the scratching resistance upon the use of ZnO-HNT nanofillers in the epoxy coating.

Xu et al. [72] carried out research on the adhesion between ZnO combined with hydroxyapatite (HAP), epoxy resin and metal substrates (mild steel panels). It was observed that the addition of ZnO-HAP improved the adhesion of the epoxy resin coating.

**Figure 2:**
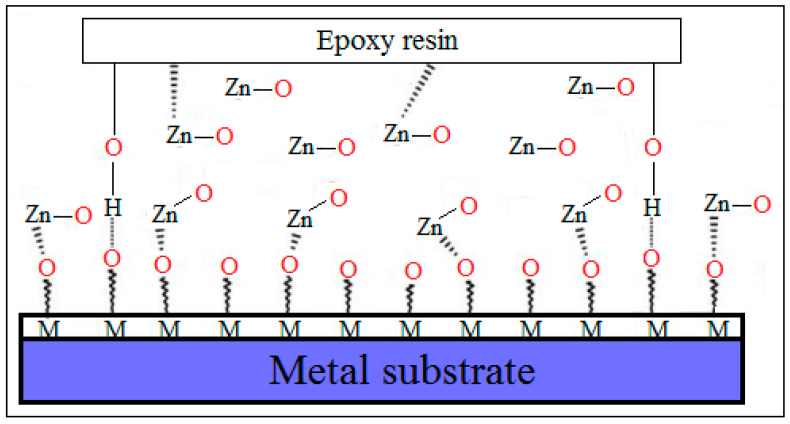
Possible mechanisms of adhesion between ZnO NPs, epoxy resin, and metal substrate. Adapted from Ref. [70].

In Figure 2 is represented the mechanism of adhesion between ZnO NPs, epoxy resin, and a metal surface. Some of the metal oxides could be hydrogen-bonded from the epoxy molecules, while others could involve interactions with Zn atoms from ZnO molecules. An improvement in the adhesive bond between the coating film and the metal substrate can occur [70].

## 2. Synthesis Methods of ZnO–Epoxy Nanocomposites

ZnO–epoxy nanocomposites can be manufactured using three methods: in situ polymerization, solution blending, and mechanical mixing. The manufacturing method must be selected considering the type of epoxy matrix, the desired properties of the final products, and the ZnO nanofillers. Table 1 shows the comparison between the various methods for the preparation of epoxy nanocomposites with nanofillers.

### 2.1. In Situ Polymerization

The uniform and homogeneous distribution of ZnO nanofillers in the epoxy matrix plays an important role in the realization of the special ZnO–epoxy composites. To obtain composites with significant strength and stiffness, it is necessary to reduce the size of the ZnO nanofillers from microns to a few nanometers.

**Figure 3:**
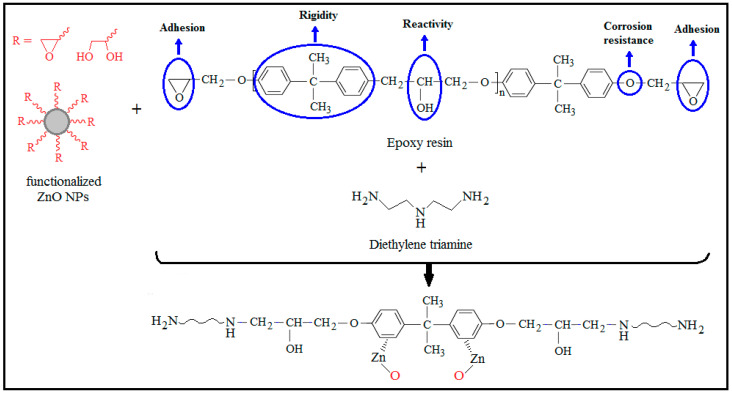
Probable mechanisms of interaction of functionalized ZnO NPs with epoxy resin in the presence of diethylene triamine. Adapted from Ref. [75].

During the curing process, an adequate dispersion of the ZnO nanofillers must be mixed with the monomer solution in order to obtain thermosetting systems [73]. Diverse reactions are possible between the surface of the ZnO NPs, the monomer solution, and the epoxy resin, depending on the functionalization of the surface and on the particle type used, all of which have an effect on the dispersion structure in the cured nanocomposite. These reactions include (i) chemical reactions between the hydroxy groups situated at the surface of the ZnO NPs with epoxides of the resin solution; (ii) the epoxy groups on the surface of the nanoparticles being attacked through the hardener molecules in order to realize covalent connection between the ZnO NPs and the resin matrix, resulting in a higher dispersion quality; (ii) strong polar interactions between the OH end-groups and the epoxy resin; and (iv) the formation of hydrogen bonding (Figure 3) [74,75].

**Figure 4:**
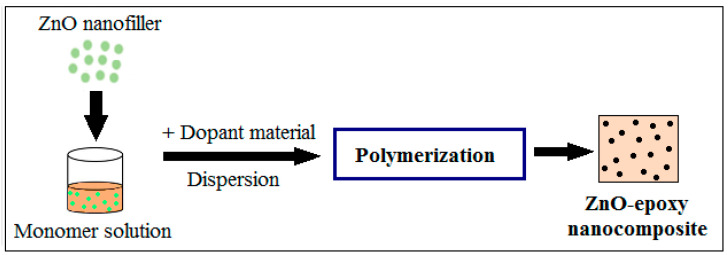
Schematic illustration of the in situ polymerization of the ZnO–epoxy nanocomposite.

The development of the ZnO–epoxy nanocomposite using in situ polymerization is schematically represented in Figure 4. The formation of a homogeneous mixture, the utilization of cost-effective materials, and the realization of complex structures are some of the advantages of in situ polymerization.

### 2.2. Solution Blending

**Figure 5:**
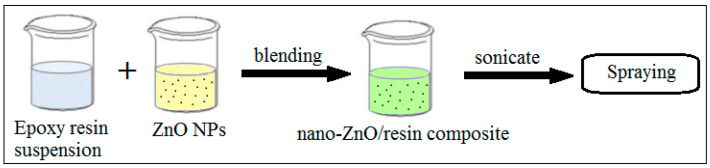
Flow chart of realization of the nano-ZnO/epoxy composite coating using the blending method. Adapted from Ref. [77].

Solution blending is a versatile, relatively cheap and simple method for obtaining ZnO–epoxy nanocomposites, and it has been used for introducing alternative properties or functions to the core material. This method involves dissolving both the epoxy resin matrix and the nanofillers in a common environmentally friendly solvent, followed by mixing, ultrasonic irradiation, or magnetic stirring, and the subsequent evaporation of the solvent to form nanocomposite particles. Adding a small amount of nanofillers to the mixture is of great importance to reduce the viscosity of the epoxy matrix, and this helps to reduce the formation of air bubbles during the stirring process in order to avoid defects in the material [76]. The procedure for obtaining ZnO–epoxy nanocomposites via solution blending is represented in Figure 5. The obtained nanocomposite can be sprayed onto metal surfaces to form a cured coating [77].

### 2.3. Mechanical Mixing

**Figure 6:**
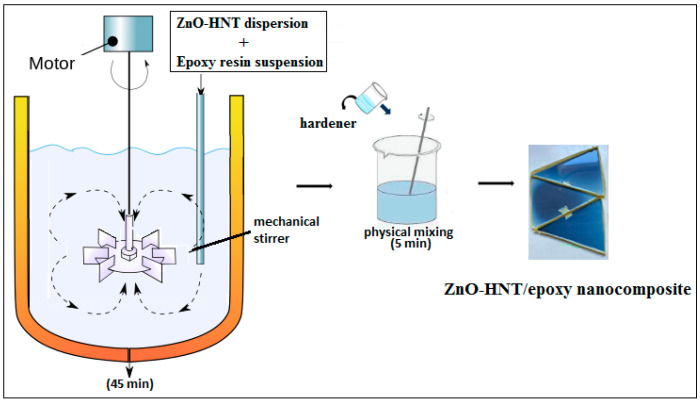
Schematic illustration of the synthesis procedure for ZnO-HNT/epoxy nanocomposite (reproduced with permission from Ref. [71]).

Mechanical mixing is yet another commonly used processing method for obtaining ZnO–epoxy nanocomposites. ZnO nanofillers are introduced into the epoxy resin and mixed with a mechanical mixer, using the force of high shearing to prevent ZnO NP agglomeration. This method is suitable for mixing miscible epoxy resins. The use of mechanical mixing can help achieve compatibility between different epoxy mixtures, improve processing efficiency, and create new materials with unique properties. ZnO–epoxy nanocomposites can be obtained by mechanical mixing using ZnO NPs and halloysite nanotubes (HNT) as nanofillers, and using epoxy resin suspension. The procedure for obtaining the ZnO–epoxy nanocomposite by mechanical mixing is shown in Figure 6.

In addition to the use of conventional mixers (which often do not provide the necessary shear forces to break up agglomerates and achieve uniform dispersion), mechanical mixing can also be achieved by other methods:

⮚ Planetary Mills

Planetary mills consist of one or more containers (milling jars) that rotate on their axes and simultaneously revolve around a central axis. This generates high shear forces and energy input. Among the different advantages of this method are the high energy input (effective in breaking down agglomerates of nanoparticles), the achievement of uniform dispersion (the combination of rotational and revolution motion ensures a more uniform distribution of nanoparticles within the epoxy resin), and the scalability of the process to industrial applications. The main disadvantages of this method are the high energy consumption, the potential for contamination, and the relatively extended milling times [78].

⮚ Three-Roll Mixers

Three-roll mixers use three horizontally positioned rollers that rotate in opposite directions and at different speeds. Material is passed through the rollers, experiencing high shear forces. The method requires the preliminary mixing of the ZnO NPs and epoxy resin, followed by multiple passes of the mixture through the rollers in order to ensure the uniform dispersion of nanoparticles. Among the advantages of the method can be listed the high shear forces (effective in breaking down nanoparticle agglomerates), the control over dispersion (as the gap between the rollers can be adjusted), and the improved mechanical properties obtained for the final composite, while its disadvantages are related to the suitability of low to moderate viscosity materials, high costs, the requirement for skilled operators, and particle size limitations (the method may not be effective for dispersing very small dimension nanoparticles) [79].

⮚ High-Speed Homogenizers

High-speed homogenizers use a rapidly rotating rotor to create intense hydraulic shear forces within the mixture, having as advantages the rapid and effective dispersion of the NPs, the precise control of the mixing parameters (such as speed and time), and versatility (they can be used for a wide range of viscosities and material types). In practice, the nanoparticles need to be pre-mixed with the epoxy resin to form a slurry, which is processed in the high-speed homogenizer, where the rotor-stator mechanism generates high shear forces. Among the disadvantages of this equipment can be mentioned the generation of significant heat (which may affect the properties of the epoxy resin or cause premature curing), equipment wear (caused by the high-speed operation), or limited particle size reduction (compared with other methods, such as the planetary mills) [80].

### 2.4. Dispersion of Nanofillers Using Ultrasound Energy

The homogeneous incorporation of nanofillers (in particular, ZnO) into epoxy resin matrices can significantly enhance the mechanical, thermal, and electrical properties of the resulting material. As an alternative mixing method, the use of ultrasound energy has proven effective in distributing ZnO fillers within the epoxy resin.

Ultrasonic mixing involves the use of high-frequency sound waves to generate cavitation bubbles in a liquid medium. When these bubbles collapse, they produce intense localized shear forces and micro-jetting effects, which help to deagglomerate and evenly disperse nanoparticles.

Nanofillers should initially be mixed with epoxy resin to form a preliminary slurry, which is subjected to ultrasonic waves using a probe-type ultrasonic processor. Parameters such as frequency, amplitude, and duration are optimized to achieve the desired dispersion quality. The advantages of this method include effective deagglomeration (as ultrasonic energy is highly effective at breaking down nanoparticle agglomerates), enhanced interfacial interaction (the cavitation effect improves the wetting and interfacial bonding between the nanoparticles and the epoxy matrix), and the scalability.

Among the disadvantages limiting its application can be noted the heat generation (ultrasonication generates significant heat, which can lead to premature curing of the epoxy resin or degradation of its properties), which requires effective cooling systems, limited penetration (especially in larger batches, the penetration of ultrasonic waves may be insufficient to ensure uniform dispersion throughout the entire volume, requiring longer processing times, batch-wise processing, or solvent addition), and energy consumption [81].

### 2.5. Stability Evaluation Methods

The stability evaluation of ZnO–epoxy nanocomposites involves a multifaceted approach, including mechanical, thermal, environmental, and dispersion quality assessments. Mechanical tests such as tensile, flexural, and impact testing, thermal analysis, and environmental tests provide comprehensive insights into the performance and durability of these nanocomposites. Additionally, dispersion quality evaluations using sedimentation tests, rheology determinations, or other analytical determinations are crucial for ensuring the uniform distribution and stability of ZnO nanoparticles within the epoxy matrix. These evaluations are essential for optimizing the properties and ensuring the long-term reliability of ZnO–epoxy nanocomposites in various applications.

⮚ Mechanical Stability Evaluation

A series of mechanical stability tests are usually applied for the evaluation of the final nanocomposite, including tensile testing (for the evaluation of ZnO nanoparticle dispersion and the strength of interfacial bonding between ZnO and the epoxy matrix, as poor dispersion can lead to stress concentrations and premature failure), flexural testing (performed in order to assess the composite’s resistance to bending and the distribution of ZnO nanoparticles within the matrix, with inhomogeneous dispersion causing localized weaknesses), and impact testing (providing insights into the toughness and energy absorption capabilities, indicating the effectiveness of nanoparticle reinforcement) [82].

⮚ Thermal Stability Evaluation

Thermal analyses are currently used for the determination of thermal stability and degradation temperatures (thermogravimetric analysis), identification of glass transition temperature (Tg) and curing behavior (differential scanning calorimetry), and to obtain information on storage modulus, loss modulus, and damping factor (dynamic mechanical analysis), reflecting a proper distribution of the nanofillers and possible interaction between ZnO nanoparticles and the epoxy matrix [83].

⮚ Environmental Stability Evaluation

Several environmental stability tests can be performed on the final nanocomposites, in order to evaluate the contribution of the nanofiller to the enhancement of the resin’s properties. Among these types of assays, the most frequently encountered are UV aging (performed by exposing the nanocomposites to UV radiation to simulate long-term environmental exposure, in order to assess resistance to photodegradation) [84], moisture adsorption tests (performed by the immersion of the nanocomposites in water or by exposing them to high humidity environments to measure moisture uptake, in order to evaluate the water resistance and the effect of moisture on mechanical and thermal properties) [85], and chemical resistance tests (performed by exposing nanocomposites to various chemicals and solvents, in order to evaluate the nanocomposites’ resistance to various environments) [71].

⮚ Dispersion Quality Evaluation

A series of analytical methods can be used for the evaluation of the dispersion quality. These include the determination of the rheological properties (determining the viscosity, shear thinning, and thixotropic behavior, providing insights into the dispersion state and interactions between nanofillers and the epoxy matrix) [74], direct observations on nanofillers within the epoxy matrix (using scanning/transmission electron microscopy) [74], phase and crystallinity determinations on the nanofillers (using X-ray diffraction) [74], or the evaluation of particle dispersion stability and particle dimensions (using methods such as sedimentary tests [86] or dynamic light scattering measurements [74]).

## 3. The Mechanical Properties of ZnO–Epoxy Nanocomposites

The mechanical properties of the epoxy composites, such as tensile strength, elongation at break, Young’s modulus, stiffness, hardness, and wear rate, are strongly influenced by the dispersion uniformity, morphology, microstructure, and concentration of nanofillers in the epoxy matrix [87,88]. Also, the reaction rate, reaction conditions, and molar ratio between nanofillers and epoxy groups are important for the formation of strong cross-linking networks, leading to the realization of epoxy systems with excellent mechanical properties [89,90].

Table 2 presents the summary of research about the mechanical properties of epoxy composites containing nano-ZnO (commercial ZnO NPs, recycled ZnO, and ZnO NPs functionalized with graphene oxide (GO), oil palm (OP), and titanium dioxide (TiO_2_)) [91,92,93,94,95,96,97,98,99,100,101]. Analyzing Table 1, it can be concluded that the mechanical performance of ZnO–epoxy nanocomposites depend on the type and concentration of the nanofiller and the epoxy resin type. It emerged from the author’s investigations that the type of functional groups grafted onto the surface of the ZnO nanofillers would provide different internal interactions, improving the mechanical properties of ZnO–epoxy nanocomposites.

**Figure 7:**
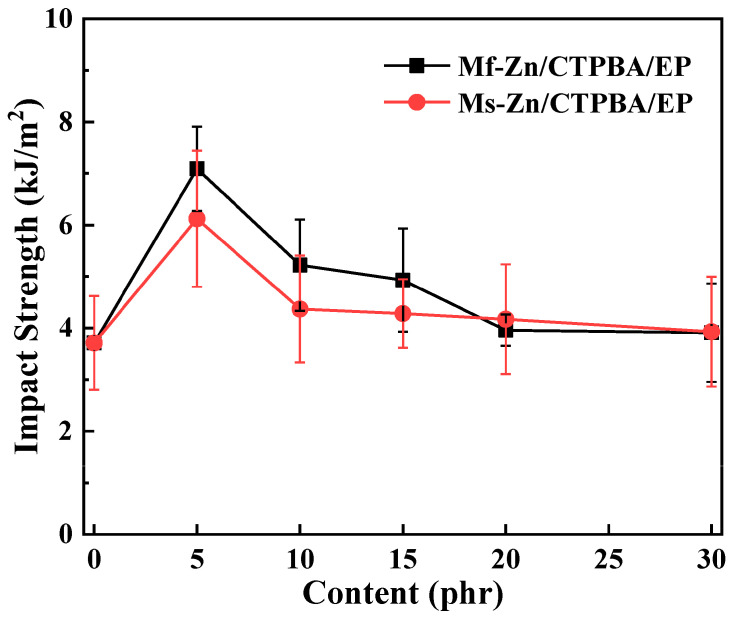
Impact strength curve of Mf-Zn and Ms-Zn on CTPBA/EP (copyright, reproduced with permission from Ref. [102]).

Lue et al. [102] investigated the effect of the addition of different ZnO powders (spherical zinc powder (s-Zn) and flake zinc powder (f-Zn)) on the impact strength of carboxyl-terminated polyester/epoxy resin (CTPBA/EP) (Figure 7). Two types of composites were realized: Mf-Zn-modified epoxy resin composites (Mf-Zn/CTPBA/EP) and Ms-Zn-modified epoxy resin composites (Ms-Zn/CTPBA/EP). Analyzing Figure 7, it can be concluded that the impact strength depends on the amount of ZnO nanofillers used. With the increase in the addition amount, the impact strength of Mf-Zn/CTPBA/EP and Ms-Zn/CTPBA/EP started to decrease. This can be due to increased defects and agglomeration when the nanofillers amount was too high. Also, the obtained result indicated that the addition of ZnO powders significantly improved the impact strength of the epoxy resin adhesive.

**Figure 8:**
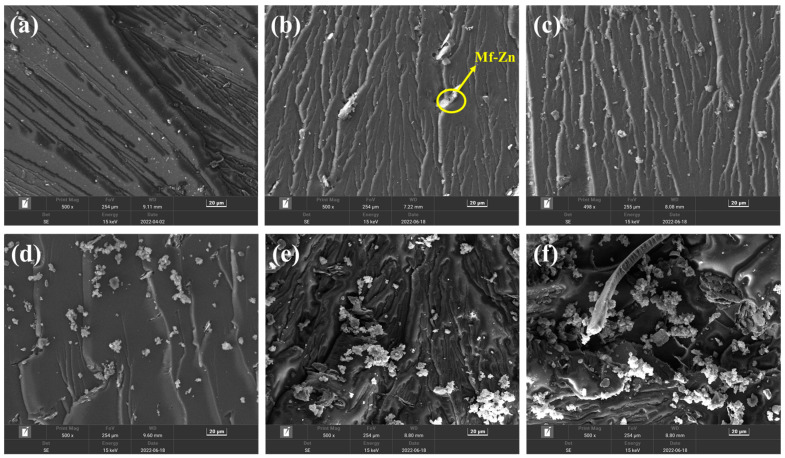
SEM images of (**a**) 0%; (**b**) 5%; (**c**) 10%; (**d**) 15%; (**e**) 20%; and (**f**) 30% of the mass of the Mf-Zn-modified CTPBA/EP epoxy resin impact section (copyright, reproduced with permission from Ref. [102]).

Figure 8 presents the SEM images of the impact fracture surface of CTPBA/EP-cured samples with various additions of Mf-Zn. In Figure 8b,c, a large number of shear deformations, cavities, crack deformations, and terminations can be observed. It was noted that the Mf-Zn particles were uniformly distributed in the resin matrix, and some particles were located at the crack tip, contributing to the improvement of the hardness.

Thipperudrappa et al. [103] demonstrated that the incorporation of 2 wt. % ZnO in epoxy resin improves the mechanical properties (flexural strength and impact strength) of the reinforced epoxy composite.

## 4. The Anti-Corrosion Performance of ZnO–Epoxy Nanocomposites

Throughout their life in the marine environment, ships, boats, and submarines are exposed to several environmental stressors. Many factors, such as the temperature, composition, and salinity of seawater, can cause damage to the materials when they are exposed for long periods [104,105]. Damaged surfaces contain holes and cracks that can grow in length and depth with exposure to aggressive environments. Although protective layers are applied to the structures, the harsh environment can cause damage to the submerged parts, as corrosion-resulting species can break the coating structure [106,107,108,109,110].

Many studies revealed that the incorporation of the ZnO nanofillers into low-cost epoxy coatings improves the anti-corrosive performance of various substrates (i.e., stainless steel, aluminum alloys, and magnesium alloys). Table 3 presents the summary of research about the anti-corrosion properties of epoxy composites/coatings containing nano-ZnO (commercial ZnO NPs, and ZnO NPs functionalized with graphene (Gr), graphene oxide (GO), silane agent ((3-glycidyloxypropyl)trimethoxysilane (GPTMS)), titanium dioxide (TiO_2_), nickel oxide (NiO), and hydroxyapatite (HAP)) [111,112,113,114,115,116,117,118].

**Figure 9:**
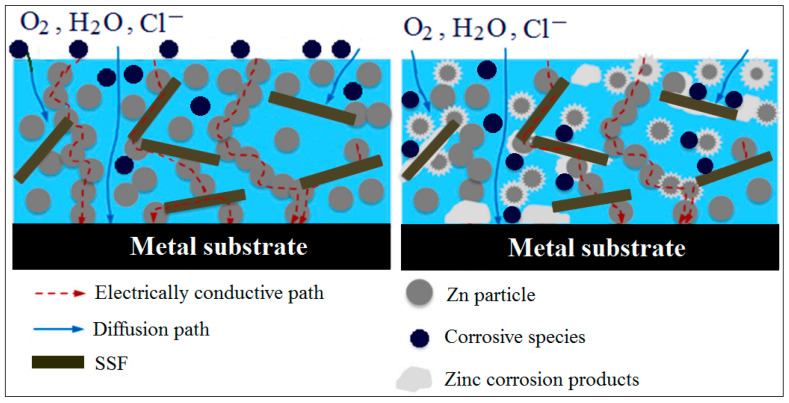
Schematic diagram of the corrosion protection mechanism of the ZRE coating with SSF. Adapted from Ref. [119].

Qi et al. [119] reported that the presence of 2.5 wt. % and 5 wt. % stainless steel flakes (SSF) could provide an improved protective barrier against corrosive environments by providing a less aggressive environment for the corrosion of zinc particles and metal substrates. A schematic diagram showing the corrosion protection mechanism of zinc-rich epoxy (ZRE) coating with SSF is illustrated in Figure 9. Zinc (Zn) corrosion begins with anodic zinc dissolution and cathodic oxygen reduction. The released Zn^2+^ ions will migrate to the cathodic sites and buffer them, contributing to the formation of Zn(OH)_2_. The moderately soluble zinc oxides/hydroxides can react with absorbed or dissolved CO_2_ from the environment in the electrolyte and Cl^−^ ions in order to form complex insoluble corrosion products in various sites. The incorporation of SSF into the ZRE coating supplies many cathodic sites for oxygen reduction, and some zinc corrosion products are expanded on the SSF surface, with fewer anodic sites (ZnO NP surface) coated with corrosion products. In this way, there is less anodic polarization of the Zn NPs and therefore the period of cathodic protection is extended. Furthermore, SSF acts as a “bridge” and increases the rate of zinc utilization by connecting multiple isolated Zn NPs into conductive percolation pathways. The presence of lamellar SSF can enhance the physical barrier effect by making it mor tortuous for the diffusion paths of corrosive species to reach the metal substrate, and at the same time, the oxidation rate of Zn NPs will be reduced (see Figure 9).

## 5. Applications of ZnO–Epoxy Nanocomposites

Since epoxy coatings are susceptible to surface abrasion, ZnO was added as nanofillers to enhance the anti-corrosive performance of the hybrid coatings [119,120,121,122,123]. ZnO–epoxy composites are used in the polymer industry as additives due to their good stability and high UV absorption [89].

Another potential application of ZnO–epoxy composites is represented by the protection of wooden objects. The hydrophobicity and color stability of wood are important properties that can easily be altered when wood is used as a raw material for outdoor products. ZnO–epoxy composites could be applied to protect the color stability of wooden objects, while also providing hydrophobicity to the surface. The composite has been demonstrated to possess superior qualities for this particular application, compared with pure epoxy [124]. ZnO–epoxy resin solutions could also be applied to glass substrates to produce a superhydrophobic coating that will reduce the adhesion of water molecules and the tensile strength [125]. ZnO NP–epoxy composites can find applications in civil and defense constructions, as surface coatings for composites, in order to increase their corrosion resistance and improve the mechanical surface behavior [126].

Liu et al. [127] reported the preparation of a ZnO/basalt composite to obtain an anti-corrosive functional coating that can be used in the marine industry. Also, Alam et al. [128] showed that the addition of 2 wt.% ZnO NPs along with the silica nanoparticles in epoxy resin increased the hardness of the coating. The nanocomposite coating presented corrosion resistance to a marine environment and it can be used in many fields of the maritime industry.

Roudpishi et al. [129] concluded that the use of 1 wt.% ZnO in epoxy resin has a significant effect on the strength of the epoxy composite and improves the critical buckling load of the glass/epoxy composites after sunlight irradiation.

**Figure 10:**
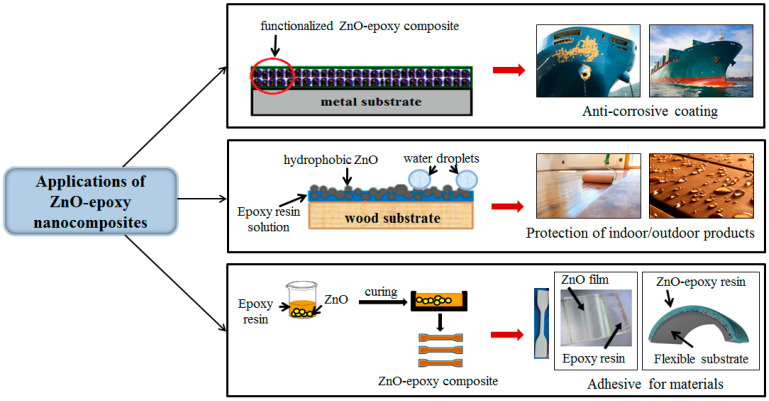
Schematic illustration of ZnO–epoxy nanocomposite applications in the construction, architecture, and naval industries.

In Figure 10 are illustrated the main applications of ZnO–epoxy nanocomposites that can be used in the construction, architecture, and naval industries.

## 6. Current Challenges and Future Research Directions

Identifying and developing epoxies that possess increased functionality remains a significant challenge within the field. Future research should focus on optimizing the concentration of the epoxy resin solution and the concentration of nanofillers to achieve superior mechanical properties. Additionally, refining the drying processes and overall composite formulation can contribute to the advancement of epoxy materials with enhanced performance.

One of the primary obstacles is the inherently weak interaction between epoxy resins and nanofillers. It is imperative to delve deeper into understanding how nanofillers influence the structural integrity of epoxy resins. By doing so, researchers can work towards enhancing the overall properties of epoxy coatings, including but not limited to corrosion resistance and thermo-chemical stability. This will require detailed studies on the interfacial interactions and the resultant effects on the composite’s microstructure and performance.

The creation of bio-based, fully recyclable epoxy resins is a promising direction for the future, aimed at improving the durability and sustainability of epoxy composites. There is a pressing need for the development of eco-friendly and economically viable epoxy materials that do not compromise on performance. Such advancements will not only contribute to environmental sustainability but also address economic concerns associated with the production and disposal of epoxy materials.

In summary, the future of ZnO–epoxy nanocomposites lies in overcoming these challenges by developing more functional epoxies, enhancing the interaction between resins and nanofillers, and creating sustainable, recyclable materials. This holistic approach will pave the way for advanced epoxy composites with superior mechanical, chemical, and environmental properties.

## 7. Conclusions

The presented work aimed to review the main characteristics and applications of nanofiller/epoxy resin composites. The mechanical and anti-corrosion performances of the composites depend on the type and concentration of the nanofillers, as well as the methods used to obtain them. This review clearly demonstrates that ZnO nanofillers are suitable candidates to increase the lifetime of epoxy resin coatings and reduce the cost of repair services. ZnO nanofillers have various advantages as anti-corrosive materials, including non-toxicity, UV degradation resistance, corrosion inhibition, and an environmentally friendly nature. Interactions between the nanofillers, the epoxy matrix, and the metal surface to improve the properties were also discussed. ZnO–epoxy resin composites have a wide range of applications, including protective coatings from corrosion and UV rays, adhesives, building materials, electronics, and the chemical industry.

This work underscores the critical importance of advancing the field of ZnO–epoxy composites, emphasizing the potential of these materials to revolutionize various applications due to their enhanced mechanical properties, adhesion strength, and corrosion resistance. The comprehensive examination of preparation methods, mechanical performance, and anti-corrosion properties of ZnO–epoxy composites offers valuable insights into the optimization of these materials for diverse industrial applications.

ZnO–epoxy composites demonstrate significant potential in various sectors, including anti-corrosion and UV-protective coatings, adhesives, the chemical industry, building materials, and electronics. The ability to tailor the properties of these composites through careful selection and modification of their components makes them invaluable for addressing specific industrial challenges.

Ultimately, this review highlights the necessity of continued research and innovation in the field of ZnO–epoxy composites. By addressing current challenges and exploring future directions, such as enhancing the interactions between epoxy resins and nanofillers and developing eco-friendly, recyclable epoxy materials, the scientific community can pave the way for the next generation of high-performance composites. This work not only contributes to the academic understanding of ZnO–epoxy systems but also has practical implications for developing advanced materials that meet the evolving demands of modern industries.

## Figures and Tables

**Table 1:** Comparison between the various methods of production of epoxy nanocomposites with nanofillers.

Methods	Advantages	Disadvantages
In situ polymerization	⮚ It allows the uniform dispersion of the nanofiller in the epoxy matrix. ⮚ The nanofiller does not always have to be exfoliated before the preparation of the nanocomposite.	⮚ Due to the attachment of epoxy chains with nanofillers, the connections between the nanoparticles are obstructed, which lowers the electrical properties of the nanocomposite. ⮚ The use of curing agents that can modify the physical properties and influence the functionality of nanocomposites.
Solution blending	⮚ Easy operation. ⮚ Low-cost method. ⮚ Ability to achieve good dispersion of the nanofiller throughout the epoxy matrix.	⮚ The use of large amounts of organic solvents that are harmful to the environment and pose safety risks. ⮚ The process may require additional steps, such as rotary evaporation, to remove solvents.
Mechanical mixing	⮚ Enhanced precision. ⮚ It can be run continuously. ⮚ Produces a homogeneous mixture of materials.	⮚ High power consumption. ⮚ It is not easy to mix nanoparticle powder into the main powder.

**Table 2:** A summary of research about the mechanical properties of epoxy composites containing ZnO nanofillers.

Nanofiller Type	Nanofiller Property	Concentration of Nanofiller (wt. %)	Epoxy Resin Type	Mechanical Tests	Results	Refs.
ZnO	Commercial ZnO	0.1, 0.3, 0.5, and 0.7	Epoxy resin	Bending strength, flexural modulus, and stiffness	Mechanical properties were influenced by the nanofiller content.	[91]
ZnO	Commercial ZnO (particle size of ~100 nm)	1, 2.5, and 5	Bisphenol A (DGEBA)	Tensile strength	Composite with 2.5 wt. % ZnO filler showed the best tensile strength.	[92]
ZnO	Commercial ZnO	0.5, 1, 2, and 5	Resole resin	Tensile and lap shear tests	ZnO filler enhanced the mechanical properties of epoxy adhesives.	[93]
ZnO	Commercial ZnO (particle size of ~10–30 nm)	0.1, 0.3, 0.5, 0.7, and 1	Bisphenol A (DGEBA)	Flexural strength and hardness tests	Mechanical properties increased up to certain ZnO filler (0.5 wt. %) and then properties gradually decreased.	[94]
ZnO	Synthesized ZnO NPs	0.1, 0.3, and 0.5	Epoxy resin	Tensile, impact, and flexural properties	Composite with 0.5 wt. % ZnO filler presented more tensile, impact, and flexural strength.	[95]
ZnO-GO	Commercial ZnO	0.001, 0.01, 0.1, and 0.5	Bisphenol-A EPON 828	Tensile strength, elongation at break, and Young’s modulus	Addition of a small amount of ZnO-GO (0.01 wt. %) improved the tensile strength and Young’s modulus of nanocomposite by 93%.	[96]
ZnO-OP	Commercial ZnO (99.8%)	1, 3, 5, and 10	Polymeric base material epoxy	Tensile, impact, hardness, and wear rate	Nanofillers improved the ductility and stiffness, and increased the impact strength of the epoxy nanocomposites.	[97]
ZnO	Recycled ZnO from spent alkaline batteries (average thickness of 35 nm)	0, 2, 6. 10, and 30	Bisphenol A	Stiffness and hardness tests	Utilization of 30 wt. % recycled ZnO NPs enhanced stiffness and hardness (82.3%) of epoxy composite.	[98]
ZnO-TiO_2_	Commercial ZnO (white powder of purity 99%)	29	Bisphenol A	Impact resistance, ductility, and hardness	ZnO-TiO_2_ reinforced the epoxy matrix and improved the adhesion and elasticity of the coating film.	[99]
ZnO	Synthesized ZnO NPs	1, 1.5, 2, 3, 4, 5 and 10	Bisphenol A	Tensile strength and breaking force	Epoxy resin with 2 wt. % ZnO presented good tensile strength.	[100]
ZnO-TiO_2_	Commercial ZnO (particle size of 150 nm)	1, 2 and 4	Bisphenol A	Tensile, flexural, and creep tests	Epoxy resin with 4 wt. % showed the highest flexural strength and good creep resistance.	[101]

**Table 3:** A summary of research into the anti-corrosion properties of epoxy composites/coatings containing ZnO nanofillers.

Nanofiller Type	Nanofiller Property	Concentration of Nanofiller (wt. %)	Epoxy Resin Type	Corrosion Test	Results	Refs.
ZnO	Commercial ZnO (diameter < 50 nm)	1	Bisphenol A (DGEBA)	Immersion in 3.5 wt. % NaCl aqueous solution	Epoxy coating incorporating ZnO NPs presented great corrosion resistance with potential applications in the marine environment; corrosion rate—6.4971 × 10^−6^ mm/year (compared with control—0.07699 mm/year).	[111]
ZnO	Commercial ZnO (diameter < 100 nm)	1, 3, 5, 7, and 10	Bisphenol A and butyl glycidyl ether	Tested in 3.5 wt. % NaCl aqueous solution, at ambient temperature	Epoxy coating loaded with 3 wt. % ZnO NPs provided the best corrosion protection on metal substrate; corrosion rate—0.00047 mm/year, compared with control—0.05268 mm/year.	[112]
ZnO	Commercial ZnO (diameter of 300 nm)	2, 3, and 4	Epoxy resin (E51)	Immersion in 3.5 wt. % NaCl solution for 6 h and 12 h	Epoxy coatings presented different corrosion resistances as a function of the immersion time.	[113]
ZnO-GPTMS	Commercial ZnO (diameter ≤ 50 nm)	1	Bisphenol A (DGEBA)	Immersion in 5 wt. % aqueous NaCl solution over 30 days	Good level of anticorrosion resistance revealed by increasing the thickness of the nanocomposite coating.	[114]
ZnO-Gr	Commercial ZnO (99.99%)	0.4	Waterborne epoxy resin	Immersion in 3.5 wt. % NaCl solution for 7 days	Utilization of ZnO enhanced the dispersibility of graphene, improving the anticorrosive performance of epoxy coatings; impedance—200,530 Ω cm^2^.	[115]
ZnO-GO	Commercial ZnO	0.001, 0.01, 0.1, and 0.5	Bisphenol-A (EPON 828)	Exposed to 3.5 wt. % NaCl for 14 days	Epoxy nanocomposite with 0.1 wt. % ZnO-GO presented the most corrosion resistance with protection efficiencies of 61% (compared with 0.01% ZnO-GO—27% and 0.1 wt. % GO—40%).	[96]
ZnO-GO	Commercial ZnO (average diameter of 50 nm)	0.1	Bisphenol A (DGEBA, EPON 828)	Immersion in 3.5 wt. % NaCl salt solution	Utilization of ZnO-GO epoxy composite enhanced the anticorrosion property of the coating; impedance—2 × 10^10^ Ω cm^2^; protection efficiency 90%.	[116]
ZnO-TiO_2_	Commercial ZnO (white powder of purity 99%)	29	Bisphenol A	Exposed to 3.5 wt. % NaCl for 28 days	Inclusion of ZnO-TiO_2_ into epoxy resin increased the durability and corrosion resistance of epoxy coatings; impedance—14,325 Ω cm^2^ after 28 days, compared with ZnO—7409 and TiO_2_—2104 Ω cm^2^.	[99]
ZnO-NiO	Synthesized ZnO NPs (powder)	1	Bisphenol A	Immersion in 3.5 wt. % NaCl solution	Incorporation of ZnO-NiO into epoxy nanocomposite improved the anti-corrosion performance of coating; corrosion rate—2.47 mpy (milli-inches per year), compared with control—43.99 mpy.	[117]
ZnO-NiO	Commercial ZnO	1	Bisphenol A	Tested in 3.5 wt. % NaCl	Utilization of ZnO-NiO in epoxy matrix enhanced the corrosion resistance of coating; corrosion rate—0.02 mpy, compared with control—13.54 mpy.	[118]
ZnO-HAP	Commercial ZnO	80, 20 and 50	Epoxy resin (E51)	Tested in 3.5 wt. % NaCl solution for 24 and 72 h	Epoxy resin coating with 65 wt. % ZnO presented the greatest anti-corrosion performance; resistance—31,432 Ω cm^2^, compared with control—688.9 Ω cm^2^, after 72 h.	[72]

## Data Availability

Data are contained within the article.
